# Biological basis of tobacco addiction: Implications for smoking-cessation treatment

**DOI:** 10.4103/0019-5545.74303

**Published:** 2010

**Authors:** R. C. Jiloha

**Affiliations:** Department of Psychiatry, G. B. Pant Hospital, Maulana Azad Medical College and University of Delhi, India

**Keywords:** Acetylcholine receptors, addiction, dopamine, nicotine

## Abstract

Tobacco use became common all over the world after discovery of Americas. Tobacco, a plant carries in its leaves an alkaloid called nicotine, which is responsible not only for several pathophysiological changes in the body but also develops tolerance to its own action with repeated use. Studies suggest that the alpha-4 beta-2 nicotine acetylcholine receptor subtype is the main receptor that mediates nicotine dependence. Nicotine acts on these receptors to facilitate neurotransmitter release (dopamine and others), producing pleasure and mood modulation. Repeated exposure to nicotine develops neuroadaptation of the receptors, resulting in tolerance to many of the effects of nicotine. Withdrawal symptoms appear on stoppage of tobacco use, which are characterized by irritability, anxiety, increased eating, dysphoria, and hedonic dysregulation, among others. Smoking is also reinforced by conditioning. Pharmacotherapies for smoking cessation should reduce withdrawal symptoms and block the reinforcing effects of nicotine obtained from smoking without causing excessive adverse effects.

## INTRODUCTION

Tobacco use is surely one of the strangest of human behaviors.[1] How is it that nearly one-third of world’s adult population regularly performs this strange act, which is necessary neither for the maintenance of life nor for the satisfaction of social, cultural, or spiritual needs; an act which is acknowledged by its adherents to be harmful to the health and even distasteful?[2] The ubiquity of tobacco use and its persistence in the face of vigorous proscriptions, and, in our own time, despite the generally agreed health risk, is a remarkable phenomenon.

Within a short span after Christopher Columbus first observed this strange behavior of smoking among the Natives of America in 1492, tobacco use spread worldwide and assumed major social, political, industrial, economic and medical importance.[3] These 500 years of tobacco’s discovery have provided ample opportunity to the commercial forces to dictate its universal availability by way of engineering addiction to tobacco among its users.[4] We, now know that this seemingly strange behavior is driven by the pharmacological effects of nicotine present both in tobacco leaves and tobacco smoke. We also know that tobacco use harms the body, causes many diseases, compromises users’ health in genera, shortens lifespan and leads to premature death.[5]

Tobacco is a plant product obtained from an important member of *Solanaceae* family of the plant kingdom.[6] Unlike other members of this family, such as tomato and potato, which have an uncontroversial nutritional role, tobacco plant carries in its leaves quantities of an alkaloid, nicotine, which gives it instead, power over man’s mind.[1] *Nicotiana tobaccum* is the main source of tobacco though most of tobacco in Northern India and Afghanistan comes from *Nicotiana rustica*.[6]

After harvesting and curing, tobacco leaves are manufactured into consumable products, which are smokeless and for smoking. Smokeless products are for chewing, snuffing and local application, while smoking of tobacco is in the form of cigarettes, cigars, *hookah, chillum, cheroot, beedis* etc.[2]

Cigarette smoking is most common both in terms of prevalence and health consequences.[1] In Indian context *beedi* smoking is more common because of economic reasons.[2] From the incandescent tip of the lighted cigarette burning at a temperature of 800°C (1600–1800°F), the smoker with each puff draws into his mouth and lungs a hot potpourri of gases and many sized particles. About 4000 chemicals have been detected in tobacco smoke while around 3000 in smokeless tobacco. Nicotine is the main chemically active constituent present in tobacco.

## NICOTINE IN TOBACCO

Nicotine is an alkaloid (1-methyl-2-[3-pyrodyl] pyrrolidine), having carbon, hydrogen and nitrogen in proportion C_10_, H_14_ and N_2_ forming a double ring like structure. In its pure state, nicotine is a colorless, volatile, strongly alkaline liquid that turns pale yellow to dark brown on exposure to air giving it a characteristic tobacco smell.[1,2] It was first isolated from tobacco leaves by Posselt and Reimanbasic in 1828 and since then it has been extensively studied.[7] It is highly toxic and potentially lethal chemical responsible for a number of patho-physiological changes in the body, and one drop of pure nicotine is sufficient to kill a dog (or a man) within minutes.[8] In the smokeless tobacco, nicotine is dissolved in the moisture of tobacco leaf as a water soluble salt while in a burning cigarette, nicotine volatilizes and is present in the smoke as free nicotine suspended on minute droplets of tar. Nicotine not only causes damaging effects, it also leads to tolerance to its own action like other dependence producing drugs.[4] It is also a gateway drug to other drugs of abuse such as marijuana and alcohol.[7]

## NICOTINE IN THE BODY

Nicotine acts on brain and other parts of the nervous system. From tobacco smoke the nicotine enters the blood stream through the lungs while nicotine in smokeless tobacco passes through the mucosal membrane of mouth and nose or the skin. Pulmonary absorption, which is the most favored and perhaps commonest, occurs in a matter of seconds. From the lungs, chemicals in the smoke are absorbed into body’s systems and carried quickly to different parts of the body. Oral, snuffs and other smokeless tobacco products are absorbed more gradually.[9] Amount of nicotine intake from one cigarette varies widely, in accordance with the smoker’s latitude for adjusting the dose level. Nicotine intake ranges from 10 mg/day to 80 mg/day, or 0.4 mg to 1.6 mg/cigarette.[10]

After absorption, nicotine travels rapidly and reaches the brain within seven seconds; it readily crosses the blood–brain barrier. This sudden burst of nicotine in the brain causes elevation of blood pressure due to stimulation of adrenal glands resulting in discharge of epinephrine. There is also sudden release of glucose and increase in respiration, heart rate, constriction of arteries and increased alertness. Many of these effects are produced through its action on both the peripheral and central nervous system. Nicotine causes release of dopamine, therefore, the psycho-active rewards occur quickly and these rewards are highly reinforced.[7] Nicotine is distributed throughout the body, mostly to skeletal muscles and brain and activates specific receptors known as cholinergic receptors.

Nicotine binds to the receptors in the brain, where it influences the cerebral metabolism. Nicotine is then distributed throughout the body. If nicotine were not absorbed quickly from the lungs, people would not take it in the form of smoke; if it were not taken up into the brain, it would not exert its psycho-pharmacological effects; if it were not rapidly metabolized and excreted, it would probably not be taken in such often-repeated doses.

## NICOTINE AND ACETYLCHOLINE

Nicotine has structural similarity to acetylcholine (Ach), which conveys information from one neuron to another. When a nerve is stimulated, the excitation is initially propagated along the nerve fibre in the form of electrical impulse and at the nerve ending; acetylcholine is released from the synaptic vesicle into the synaptic cleft, which stimulates acetylcholine receptors in the next neuron, and the neurotransmitter is used as the messenger to pass on the information carried in the nerves. This way messages are carried from the body to the brain, from the brain to the body and between different parts of the brain and spinal cord.[2]

Acetylcholine is involved in systems concerned with mental and physical arousal, learning and memory and several aspects of emotion. There are also other receptors for acetylcholine in the body, apart from the ones at synapses. They are also found at junction of nerve and muscles and nerves and certain glands.

**Figure 1:**
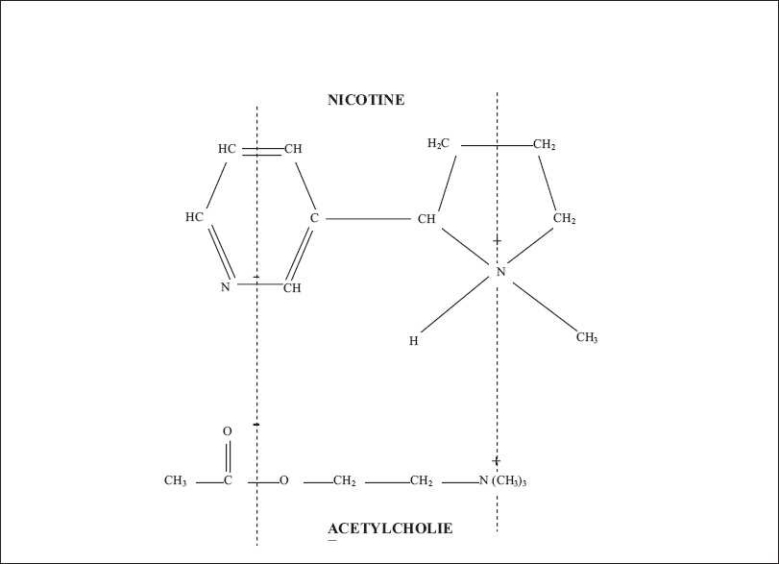
The structure of nicotine and acetylcholine

Acetylcholine receptors respond only to acetylcholine as they recognize acetylcholine molecule by the position of two electrical charges, one positive and one negative, located at certain sites of the molecule [Figure 1]. The distance between these two charges is always the same and corresponds with two equally spaced and oppositely charged sites on the receptor. The acetylcholine molecule is attracted to the acetylcholine receptor and then fits snugly into it by virtue of their mutually satisfying configuration. In the nicotine molecule the positive charge on the ammonium head and the negative charge on pyridine ring are just the same distance apart as they are in the acetylcholine molecule. This structural similarity makes nicotine molecule to interact with acetylcholine receptors.

Physiologically these receptors respond to the natural transmitter acetylcholine, however, nicotine also activates them but, unlike acetylcholine, nicotine remains for much longer and as a consequence a proportion of nAChRs are desensitized. Repeated exposure to nicotine leads to an increase in the number of nAChRs in the brains of laboratory animals and human smokers. The activation of nAChRs by nicotine can have a number of consequences in the recipient nerve cells, including the release of various other neurotransmitters.

## NEUROBIOLOGY OF NICOTINE ADDICTION

### Nicotine acetylcholine receptors

Acetylcholine receptors throughout the body are traditionally classified as nicotine receptors (those that respond to nicotine) and muscarine receptors (those that respond to muscarine). The ability of nicotine to combine with acetylcholine receptors means that it can exert actions like acetylcholine at all synapses where nicotine acetylcholine receptors (nAChRs) are present and can trigger impulses down postsynaptic nerve fibres, resulting in effects that otherwise occur only when acetylcholine is released following stimulation of the pre-synaptic nerves. Synapses involving acetylcholine are very widespread in the body, affecting systems ranging from the cardiovascular to the psychological and also interacting with other transmitter systems showing that nicotine has multifarious actions. Electrical excitation of a nerve produces not just one impulse but a whole train of impulses. This multiplicity imposes the requirement at the synapse that the combination of transmitter with the receptor must be quickly reversed between each impulse, leaving the receptor free to combine with the next pack of acetylcholine released.

Nicotine binds to the acetylcholine receptors (nAChRs) in the brain and influences the cerebral metabolism by stimulating these receptors. The stimulation of presynaptic nAChRs on the neurons increases the transmitter release as well as the metabolism. Chronic administration of nicotine results in desensitization and inactivation of nAChRs[11,12] with subsequent up-regulation of nAChRs sites.

Cholinergic receptors are concentrated in the midbrain areas, such as mid-brain tegmentum, the striatum, nucleus accumbens (NAc) and the ventral tegmentum[13] as well as in muscles, adrenal glands, the heart and other organs. These receptors are normally activated by acetylcholine. Besides binding to acetylcholine receptors (nAChRs) nicotine also binds to the cholinergic receptors in the autonomic ganglia, adrenal medulla, chemoreceptors of the carotid bodies and aortic body and neuromuscular junction. The specific sites of binding in the brain are the hypothalamus, thalamus, midbrain, brainstem and cerebral cortex. Nicotine also binds to the receptors in nigrostriatal and mesolimbic dopaminergic neurons. As and when dopaminergic receptors are stimulated, they release acetylcholine, norepinephrine, dopamine serotonin, vasopressin, growth hormone and ACTH. Nicotine is one of the most potent stimulants of the midbrain dopamine reward pathway.[14–16] Nicotine acts on locus coeruleus regulating vigilance, arousal, concentration and stress reactions making the tobacco users more alert. Owing to the interaction between nicotine and neuronal high-affinity nicotine acetylcholine receptors (nAChRs), nicotine affects learning, memory and other functions.

Nicotine also alters the function of some of the neurotransmitters implicated in the pathogenesis of some of the major psychiatric disorders. These include dopamine, norepinephrine, serotonin (5-HT), glutamate, gamma-aminobutyric acid (GABA) and endogenous opioid peptides.[17,18] These effects could be presynaptic, preterminal, or cell body nicotine receptors, rather than mediated through neurotransmission wherein presynaptically released acetylcholine acts on postsynaptic, junctional nAChRs to cause neuronal firing.[19]

The cholinergic receptors are relatively large structures consisting of several components known as subunits. Nicotine receptors are composed of 12 subunits in mammalian brain, 9 alpha subunits (alpha_2_ to alpha_10_) and 3 beta subunits (beta_2_ to beta_4_), which play the central role in autonomic transmission.[20] The AChRs complex is composed of 5 subunits and is found both in peripheral and central nervous system.[21] Different combinations make different receptors, which vary in terms of affinity and localization within the brain.[7] The most abundant receptor subtypes in the brain of humans are alpha_4_–beta_2_, alpha_3_–beta_4_ and alpha_7_ (homomeric).[4] The beta subunit has role in nicotine addiction. The alpha_4_–beta_2_ subunit combination has greatest sensitivity to nicotine.[7] In mice, knocking out beta_2_ subunit gene eliminates the behavioral effects of nicotine, including self-administration.[22] Reinserting the beta_2_ subunit gene into VTA of a beta_2_ knocked out mouse restores behavioral responses to nicotine.[23] The alpha_3_–beta_4_ and alpha_7_ (homomeric) receptor subtypes mediate the cardiovascular effects of nicotine.[24] The alpha_7_ subtype is also thought to be involved in rapid synaptic transmission and may play a role in learning[25] and sensory gating.[26]

Repeated inhalation of tobacco generates boli of nicotine delivered into the brain, superimposed on a relatively stable level of plasma nicotine maintained by the smoker throughout the smoking day. This basal level of nicotine keeps a proportion of nAChRs in a desensitized state, while the remaining are available for activation by nicotine boli, if appropriate concentrations are achieved.[7] This way smokers manipulate their plasma nicotine profile to achieve balance desensitization versus activation. When a smoker is asleep, plasma level of nicotine decreases and the nicotine receptors gradually recover their active function. In the morning, a smoker has a greater number of active nAChR sites (up-regulation) contributing to withdrawal symptoms and craving. It is, therefore, the first cigarette of the day is, most satisfying, as overnight abstinence allows a substantial recovery from nAChRs desensitization. Post-mortem findings in smokers brain show increased number of nAChR binding sites.[27] With repeated exposure to nicotine, there is neuroadaptation to some of the effects of nicotine,[28] leading to an increase of nAChRs to up-regulate the nicotine-mediated desensitization. This desensitization plays role in nicotine tolerance and dependence. Craving and withdrawal symptoms begin in chronic smokers when previously desensitized alpha_4_–beta_2_ nAChRs become unoccupied and recover to a responsive state during the period of abstinence i.e. night sleep.[29]

Electrophysiological studies demonstrate that nicotine agonists stimulate the release of GABA from rodent brain and this release are Ca^2+^-dependent.[30,31] The actions of nicotine on ventral tegmental GABAergic innervation, which modulates the mesolimbic dopamine excitability, have been studied.[32] Nicotine was found to increase firing rate of dopamine and non-dopamine neurons, while the former was more vigorous. These findings suggest that nicotine stimulates the firing rate of dopaminergic neurons of VTA and also the GABAergic neurons, which may be an important target for the effects of nicotine on the central nervous system.

Acute nicotine administration stimulates the release of noradrenalin (NA) in the different parts of the brain, primarily at locus coeruleus level.[33] Chronic nicotine administration decreases the concentration of 5-HT in the hippocampus,[34] because it is associated with selective increase in the density of 5-HT1A receptors in this area. Hippocampus receives serotonergic innervation from the median raphe nucleus. Suppression of 5-HT release brings about anxiolytic response to nicotine microinjection into the dorsal hippocampus.[35] The effects of nicotine on 5-HT are difficult to dissociate from those on dopamine neurons. Increased exposure to stressful stimuli is likely to increase the desire to smoke as reported by smokers.[36] The effects of nicotine withdrawal on dopamine release in the brain may be exacerbated by exposure to stressful stimuli and may underlie the role of stress as a factor in tobacco smoking, as well as the role of nicotine on reducing the effects by acting on 5-HT neurons within the hippocampus.

Animal studies suggest commonalities between nicotine withdrawal and opiate abstinence syndrome. Nicotine stimulation induces the release of endogenous opioid peptides in various brain regions resulting in over-activation of opiate receptors resembling opiate dependence. Abrupt termination of nicotine stimulation may precipitate an opiate abstinence like syndrome.[37] A recent study suggests that cotinine, a metabolite of nicotine stimulates nicotine receptors to evoke the release of dopamine (DA) in a calcium-dependent manner from super-fused rat striatal slices.[27]

Advanced neuroimaging technique shows dramatic effect of tobacco smoking on the brain of awake human beings. On positron emission tomography (PET), it was found that cigarette smoking decreases the levels of monoamineoxidase (MAO), which is responsible for breakdown of dopamine. Decrease in the levels of MAO-A and MAO-B results in increase of dopamine levels.[38–40]

### Biology of nicotine reinforcement: Dopamine and reward pathways

Brain imaging studies show that nicotine acutely increases activity in prefrontal cortex, thalamus and visual system consistent with activation of cortico-basal ganglia-thalamic brain circuits.[41] Nicotine is a powerful reinforcing agent in both animals and humans. The mesolimbocortical dopamine system consists of neurons with cell bodies localized in ventral tegmental area (VTA) and axon projections to nucleus accumbens (NAc) and medial prefrontal cortex. Nicotinic receptors concentrated in VTA and NAc activate the mesolimbic dopamine system, which is responsible for reinforcing behavior like other dependence producing drugs.[42] VTA and its projections to NAc are involved in reward and mediate the reinforcing actions of drug abuse.[43] Nicotine stimulates the release of dopamine in the pleasure circuit and increases extracellular level of dopamine in NAc.[44–46] Lesions of mesolimbic dopamine neurons attenuate nicotine self-administration in rats.[47] It also attenuates locomotor stimulant effect of systemically administered nicotine.[48] Stimulation of central nAChRs by nicotine results in the release of a variety of neurotransmitters in the brain, most importantly dopamine in the mesolimbic area, the corpus striatum and the frontal cortex. Of importance are the dopaminergic neurons in the VTA of the midbrain and release of dopamine in the shell of NAc, which is critical in drug-induced reward.[49] Other neurotransmitters, including norepinephrine, acetylcholine, serotonin, gamma-amino-butyric acid (GABA), glutamate and endorphins, are released as well, mediating various behaviors of nicotine [Table 1].[44]

**Table 1:** Neurotransmitters and their behavioral effects

Neurotransmitter	Behavioral effect
Dopamine	Pleasure, appetite suppression
Norepinephrine	Arousal, appetite suppression
Acetylcholine	Arousal, cognitive enhancement
Glutamate	Learning, memory enhancement
Serotonin	Mood modulation, appetite suppression
Beta-endorphin	Reduction of anxiety and tension
Gamma-amino-butyric acid (GABA)	Reduction of anxiety and tension

Release of neurotransmitters occurs via modulation by presynaptic nAChRs along with the direct release.[50] Dopamine release is facilitated by nicotine-mediated augmentation of glutamate release and long-term treatment by inhibition of GABA release.

### Genetics of tobacco addiction

CYP2A6 is an enzyme responsible for the majority of inactivation of nicotine in humans; it is also responsible for activating tobacco-related precarcinogens such as the nitrosamines. A common genetic defect in nicotine metabolism decreases smoking. Genetic variation in CYP2A6 gene may protect individuals from becoming nicotine-dependent smokers. Mimicking this gene defect by inhibiting CYP2A6 decreases nicotine metabolism.[51] Cigarette smoking like other behaviors shows evidence of heterogeneity. Dopamine transporter (DAT) gene (SLC6A3) encodes protein that regulates the synthetic levels of dopamine in the brain and leads to addictive behavior.[52] It is expected that recent advances in molecular biology, including the completion of draft sequence of the human genome may help in identifying gene markers that predict a heightened risk of using tobacco to increase our understanding of nicotine dependence.[53]

### Learning and memory

Nicotine plays a potential role in cognitive enhancement,[54] while the earlier studies reported improvement in learning and performance with nicotine; in the recent studies nicotine has been shown to produce a sort of place preference in rats and mice.[55,56] The self-administration of nicotine has also been demonstrated.[20,57,58] In rodents, nicotine has anxiolytic-like action on various behavioral tests namely the mirror chambered, the elevated plus maze, the two compartment light dark transition test[59] and fear-potentiated startle.[60] Nicotine and nicotine agonists improve performance in a variety of cognitive tasks by animals with basal forebrain lesion.

### Tolerance and dependence

Tolerance to nicotine and onset and persistence of withdrawal on cessation of nicotine treatment has been extensively studied in experimental animals.[61] Chronic exposure to nicotine increases high affinity binding of nicotine agonists to brain tissue and induces chronic tolerance to many of the drug’s behavioral and physiological effects.[62] The increase in receptor number (upregulation) has been interpreted as compensation for agonist-induced desensitization of nAChRs, and this prolonged desensitization has been viewed as a mechanism for chronic tolerance to nicotine.[63] Acute and chronic nicotine administration in experimentally naïve rats depresses locomotor activity.[64]

### Psychoactive effects of nicotine and nicotine withdrawal

Nicotine in humans induces stimulation and pleasure and reduces stress and anxiety. Nicotine use modulates the level of arousal and controls mood in daily life. Smoking may improve concentration, reaction time and performance of certain tasks. On stoppage of smoking, nicotine withdrawal symptoms emerge, which include irritability, depressed mood, hedonic dysregulation, restlessness, anxiety, difficulty concentrating, increased hunger, insomnia and craving for tobacco.[4] Relative deficiency of dopamine release following long-standing exposure accounts for mood disturbances, anhedonia and craving for tobacco that may persist for long time after quitting. The pharmacological basis of nicotine addiction can be viewed as the combination of positive reinforcement, such as enhancement of mood or functioning, as well as avoidance of negative consequences of prior drug use – the relief of withdrawal symptoms.

### Conditioning behavior in nicotine addiction

Drug taking is a learned behavior occurring due to conditioning, reinforced by consequences of pharmacological actions, and association with specific moods, situations, or environmental factors with the rewarding effects of the drug. Respiratory sensory cues of tobacco smoking represent a conditioned reinforcement that play an important role in smoke intake, craving and rewarding effects.[55,65]

## MECHANISM OF PHARMACOTHERAPY FOR SMOKING-CESSATION

Pharmacological effects of nicotine play a crucial role in tobacco addiction, and pharmacotherapy has to address this component of tobacco dependence. A pharmacological treatment for smoking cessation should both block the positive reinforcing effects of nicotine and prevent or reduce the development of withdrawal symptoms. Pharmacotherapy should also target the receptor subtypes involved in nicotine addiction without affecting the receptors that, it activated, would produce unwanted adverse effects.

FDA (Food and Drug Administration) of USA approved the medications used for smoking-cessation, which include nicotine replacement therapy (NRT) (transdermal patch, gum, nasal spray, inhaler and lozenges); *bupropion* and varenicline. *Nortriptyline* and clonidine, though not approved by the FDA, are clinically effective in smoking-cessation.[66]

### Nicotine replacement therapy

Nicotine replacement therapy (NRT) acts in several ways – it relieves craving and withdrawal symptoms, which are relieved with relatively low blood nicotine levels, and causes positive reinforcement for arousal and stress relieving.[67] Nicotine desensitizes alpha_4_–beta_2_ nAChRs resulting in reduced effects of cigarette smoking.

### Non-nicotine agents

#### Antidepressant agents

*Bupropion* blocks dopamine reuptake and, to a lesser extent, norepinephrine reuptake and it has some nicotine receptor-blocking activity.[68] *Bupropion* increases dopamine and norepinephrine blood levels, similar to the effect of nicotine on these neurotransmitters. In rats, *bupropion* in low doses blocks the rewarding effects of nicotine. The blockade of nicotine receptors could contribute to reduced reinforcement from a cigarette in the case of a lapse. *Nortriptyline* is a norepinephrine reuptake blocker and as such simulates noradrenergic actions of nicotine in the brain.

#### Clonidine

It is an alpha_2_ -adrenergic receptor agonist and reduces sympathetic neural outflow resulting into sedation, anxiolysis, potential hypotension, bradykinesia and dry mouth. Calming and anxiolytic effect helps in smoking cessation, particularly in those who are very anxious while quitting smoking.[69]

#### Varenicline

It is an analog of plant alkaloid, cytisine, reported to have some benefit in smoking cessation. Varenicline has high and selective activity at alpha_4_–beta_2_ receptor. It is a partial agonist at this receptor *in vivo* producing lesser response than that of nicotine (30–60%) but also blocks the effect of any nicotine added to the system. Thus, varenicline maintains a moderate level of dopamine release, which reduces craving and withdrawal symptoms during abstinence. It also blocks the reinforcing effects of nicotine obtained from cigarette smoke in the case of relapse.[70]

#### NicVAX

It works by stimulating the immune system to make antibodies that bind to nicotine molecules, making them too big to cross the blood–brain barrier and preventing them from reaching nicotine receptors and triggering the pleasure sensation that smokers and users of nicotine experience and become addicted to. Data from pre-clinical trials suggest that the injectable vaccine would be effective not only in helping people quit smoking but also in relapse prevention because the nicotine antibodies last a long time.[71]

Tobacco addiction, like other addictions, is a complex process involving the interplay of pharmacology, conditioned factors, personality and social setting. Therefore, the ideal treatment for tobacco-cessation involves a comprehensive approach that addresses all major issues of tobacco addiction both pharmacological and nonpharmacological.
